# The Provincial Medical Schools

**Published:** 1896-09-05

**Authors:** 


### The Provincial Medical Schools.

### MEDICAL STUDY IN THE PROVINCES.

The great provincial towns have done well to establish med:cal schools in their midst. Not only have hey utilised the clinical material in their great hospitals and have given to the inhabitants of their respective districts opportunities for medical study, but they have also secured to themselves the permanent residence among them of men of the highest professional rank, such as the stimulus of a school alone can make and the attraction of a school can alone retain.

We do not say that the students' opportunities for clinical study are quite the same as, or quite equal to, those offered by such a vast emporium of strange diseases as London is. But while we think a provincially educated man would do well to spend at least some time among the London hospitals, we feel sure that some of the provincial towns have in the equipment of their colleges and in their appliances for teaching surpassed many of the London medical schools. Especially is this the case where medicine is but a department in a great college. Much more than is the case in London do the great provincial colleges, such as the Owens College in Manchester, the University College in Liverpool, Mason College in Birmingham, and the Yorkshire College in Leeds, become the centres round which the intellectual life of these towns revolves, all of which is of the utmost advantage to the students who are attached to them.

At some of these colleges the scientific side of the medical education is exceedingly well carried out. First in this respect should come Cambridge, not merely from the excellence of its teaching, but from the fact that the students are attached to no one college, and tend much less to become exclusively *medical* students, and next because the knowledge of science there imparted is not medical science only, but is the general scientific grounding required for the natural science Tripos given irrespective of the fact that the student is ultimately going to devote himself to medicine. Next to Cambridge we may fairly place Owens College, Manchester, but the other places we have named follow in very close succession, and at any of the larger of these colleges the student has first-rate opportunities for obtaining a sound medical education. It should also be noted that at Newcastle-on-Tyne, and at the colleges connected with the Victoria University, there are opportunities for obtaining a degree which do not exist in London. In connection with some of the colleges there are very fine and admirably fitted hospitals, such as those at Liverpool, Leeds, and Newcastle, which give very large opportunities for clinical study, and most of them being situated in large manufacturing towns the surgical experience gained there is very large.

List of Provincial Medical Schools, Giving the Cost of Education at Each.

| Name of Medical School. | Fee for Lectures for Conjoint Board Examination and for Medical and Surgical Practice in the Hospitals. | Notes. |
|---|---|---|
| Cambridge University ... ... | The total expenses at Cambridge, including residence, lectures, etc., are stated to be at least £200 per annum; but are lessened in many cases by college scholarships, which are fairly numerous. | We give no details as to the fees at Cambridge, as it is usual to take only one portion of the course there, the rest of the time being passed in London. |
| University of Durham College of Medicine, Newcastle-on-Tyne. | £99 10 0 | In addition to this £2 2s. must be paid yearly for the first three years. Fees for lectures only £73 10s. on entrance, or £84 in two instalments, or £89 5s. in three instalments. |
| Owens College (Victoria University), Manchester. | £112 0 0 | |
| University College (Victoria University), Liverpool. | £102 0 0 | |
| Yorkshire College (Victoria University), Leeds. | £115 10 0 | |
| Mason College, Birmingham ... | £112 0 0 | The fees for lectures may be paid in two annual instalments. |
| University College, Bristol ... | £105 0 0 | The fees for lectures may be paid in two annual instalments. |
| Sheffield School of Medicine ... | — | No composition fee is given, some of the courses being taken at the Firth College. Fee for Hospital Practice, £45 in one sum. |
| University College of South Wales and Monmouthshire, Cardiff. | — | Fees for lectures only £47 10s., or £50 in two yearly instalments. (For a portion of the course only.) |

